# Keep your eyes on the prize: Tackling breakthrough COVID-19 in MS patients

**DOI:** 10.1177/13524585211057511

**Published:** 2021-11-16

**Authors:** Zoé LE van Kempen, Joep Killestein, Hans-Peter Hartung

**Affiliations:** Department of Neurology, Amsterdam University Medical Centers, Vrije Universiteit, Amsterdam, The Netherlands; Department of Neurology, Amsterdam University Medical Centers, Vrije Universiteit, Amsterdam, The Netherlands; Department of Neurology, Heinrich Heine University Düsseldorf, Düsseldorf, Germany/Brain and Mind Centre, The University of Sydney, Sydney, NSW, Australia/Department of Neurology, Palacky University Olomouc, Olomouc, Czech Republic

During the COVID-19 pandemic, the international multiple sclerosis (MS) community has gone to great lengths to answer the many questions arising from the pandemic with regard to the MS population. Together, we have set up (inter)national registries, large observational cohorts, and prospective trials with the aim of understanding and tackling COVID-19 in people with MS and adapt disease treatment. COVID-19 MS-related research has, thus far, focused on three main topics: (1) defining risk factors for a more severe course of COVID-19, (2) understanding immunity after either infection and/or SARS-CoV-2 vaccination, and (3) exploring the impact of disease modifying treatments on SARS-CoV-2 vaccination.

Many reports have been published describing the course of COVID-19 in MS patients. Large national cohorts were collected and interrogated in the United States, Italy, and France among others.^1–3^ An international cohort encompassing 1,683 confirmed COVID-19 cases in MS patients found that older age, progressive MS-phenotype, higher disability, comorbidities and anti-CD20 therapies were risk factors for a more severe course of COVID-19.^4^ Other disease modifying therapies do not seem to carry an increased risk of a worse outcome of COVID-19 in MS patients.^1–4^ Still, for all patients with MS, reaching protective immunity against SARS-CoV-2 is of utmost importance.

Infection with SARS-CoV-2 induces an immunity cascade operated by both the innate and adaptive immune system. Unfortunately, SARS-CoV-2 is very good at slowing the early interferon-related innate immune response, resulting in a delay of priming T cells and formation of antibodies, which is associated with a poor outcome.^5^ The adaptive immune reaction to SARS-CoV-2 consists of coordinated SARS-CoV-2 specific T and B cell responses and a humoral response with formation of antibodies against various SARS-CoV-2 targets such as spike and nucleocapsid. The immune memory established after SARS-CoV-2 infection encompassing antibodies, memory B cells and CD4+ and CD8+ memory T cells changes over time.^5^ Although antibody titers can decrease, SARS-CoV-2 specific memory B cells increase in numbers at first, then plateau and remain responsible for a continued humoral response.^6,7^

As MS is an immune system–related disease, and therapies are directed to induce immune modulation or suppression, immunity after SARS-CoV-2 infection or vaccination deserves extra attention.

In this issue of *Multiple Sclerosis Journal*, Bsteh et al. report on the humoral response after polymerase chain reaction (PCR) confirmed symptomatic SARS-CoV-2 infection in 125 patients with MS. In their cohort, patients were selected through the Austrian MS-COVID-19 registry. Antibodies against the S1 domain of the spike protein of SARS-CoV-2 were measured between 1.5 to 13.7 months after infection. About 76% of patients had seroconverted at the time of sampling. Neither seropositivity nor antibody titers were associated with age, comorbidities, MS course, expanded disability status scale (EDSS), or COVID-19 severity. Regarding non-induction disease modifying therapies, seroconversion rate and antibody titers were lowest in patients on anti-CD20 therapies (seroconversion 12/22, i.e. 55%) and fingolimod (seroconversion 11/16, i.e. 69%). Six patients in this cohort were treated with either cladribine or alemtuzumab and underwent seroconversion in 67%. In 103 patients, lymphocyte counts were available, sampled 3 months to 2 weeks before positive SARS-CoV-2 PCR. Lymphopenia or grades of lymphopenia were not associated with seroconversion; however, in seven patients with ⩾ grade 3 lymphopenia, only three patients seroconverted.

The most important finding of the study of Bsteh et al. is the diminished humoral response in patients on immunosuppressive therapies such as fingolimod and anti-CD20 therapies. The percentage of seroconversion after COVID-19 in patients using fingolimod is in agreement with other studies reporting between 67% and 79% conversion.^8,9^ The authors suggest a possible difference in humoral response after vaccination and infection in patients on fingolimod, as a strikingly low rate of seroconversion after vaccination has been reported (4%, 1/26).^10^ However, higher rates of seroconversion after vaccination in patients on fingolimod (63%–67%) have also been noted.^11,12^ These differences could be explained by various timing of antibody sampling in these studies or differences of assay sensitivity, especially for low titer antibodies.

The finding of decreased seroconversion in ocrelizumab- and rituximab-treated patients in the cohort reported by Bsteh et al. is in agreement with other studies reporting on SARS-CoV-2 seroconversion after infection or vaccination (38%–52%).^8,9,11,13^ Despite the reduction of seroconversion in patients with MS on anti-CD20 therapies, there remains a robust T cell response after SARS-CoV-2 vaccination which is likely to provide at least some form of protective immunity.^13,14^ The most important question that remains is to what extent are patients with tempered humoral responses after SARS-CoV-2 vaccination and/or infection protected from (severe) symptomatic COVID-19?

In an Israeli study of 152 hospitalized individuals with breakthrough COVID-19 despite complete vaccination, 40% had some form of immunosuppression (7% were treated with anti-CD20 therapies).^15^ Of the patients on anti-CD20 therapies (*N* = 10), 50% had a poor outcome (defined as mechanical ventilation or in-hospital death) versus 25% of the entire cohort. Unfortunately, no pre-breakthrough SARS-CoV-2 antibody titers were available; therefore, no conclusions could be made regarding the possible role of absent antibodies in breakthrough disease.

A recent study based on UK data sets of the general population reported on re-infections > 90 days after COVID-19 in patients with and without antibodies after the primary infection.^16^ Of 224 patients without antibodies, 0.89% experienced a re-infection comparable to a 18/2087 (0.86%) of individuals with detectable SARS-CoV-2 antibodies after the initial infection. Unfortunately, no details regarding the cause of lack of antibodies or disease severity of re-infection were reported.

Although it is remarkable how many data were gathered in the last year and a half, now is the time to take COVID-19 research a step forward and define the remaining research topic(s) on which to focus next. Internationally, we should gather as much information as we can regarding SARS-CoV-2 re-infections and breakthrough COVID-19 after vaccination using our well-set-up MS registries. The influence of therapies, prior antibodies, and possible additional booster vaccinations should be assessed on (severity of) re-infections and breakthrough COVID-19. In this way, we can move forward in tackling COVID-19 in our MS population.
